# Effects of a clinic-based reproductive empowerment intervention on proximal outcomes of contraceptive use, self-efficacy, attitudes, and awareness and use of survivor services: a cluster-controlled trial in Nairobi, Kenya

**DOI:** 10.1080/26410397.2023.2227371

**Published:** 2023-08-18

**Authors:** Jasmine Uysal, Sabrina C. Boyce, Chi-Chi Undie, Wilson Liambila, Seri Wendoh, Erin Pearson, Nicole E. Johns, Jay G. Silverman

**Affiliations:** a Predoctoral fellow, Center on Gender Equity and Health, Department of Medicine, Division of Infectious Disease and Global Public Health, University of California, San Diego, CA, USA.; b Postdoctoral fellow, Center on Gender Equity and Health, Department of Medicine, Division of Infectious Disease and Global Public Health, University of California, San Diego, CA, USA; c Senior Associate, Population Council, Nairobi, Kenya; d Associate, Population Council, Nairobi, Kenya; e Global Lead for Gender & Inclusion, International Planned Parenthood Federation, London, UK; f Research Scientist, Center on Gender Equity and Health, Department of Medicine, Division of Infectious Disease and Global Public Health, University of California, San Diego, CA, USA; g Data Analyst, Center on Gender Equity and Health, Department of Medicine, Division of Infectious Disease and Global Public Health, University of California, San Diego, CA, USA; h Professor of Medicine and Global Public Health, Center on Gender Equity and Health, Department of Medicine, Division of Infectious Disease and Global Public Health, University of California, San Diego, CA, USA

**Keywords:** reproductive coercion, family planning, intimate partner violence, contraceptives, intervention

## Abstract

This study was undertaken to evaluate the effect of a reproductive empowerment contraceptive counselling intervention (ARCHES) adapted to private clinics in Nairobi, Kenya on proximal outcomes of contraceptive use and covert use, self-efficacy, awareness and use of intimate partner violence (IPV) survivor services, and attitudes justifying reproductive coercion (RC) and IPV. We conducted a cluster-controlled trial among female family planning patients (*N* = 659) in six private clinics non-randomly assigned to ARCHES or control in and around Nairobi, Kenya. Patients completed interviews immediately before (baseline) and after (exit) treatment and at three- and six-month follow-up. We use inverse probability by treatment weighting (IPTW) applied to difference-in-differences marginal structural models to estimate the treatment effect using a modified intent-to-treat approach. After IPTW, women receiving ARCHES contraceptive counselling, relative to controls, were more likely to receive a contraceptive method at exit (86% vs. 75%, *p* < 0.001) and had a significantly greater relative increase in awareness of IPV services at from baseline to three- (beta 0.84, 95% CI 0.13, 1.55) and six-month follow-up (beta 0.92, 95% CI 0, 1.84) and a relative decrease in attitudes justifying RC from baseline to six-month follow-up (beta −0.34, 95% CI −0.65, −0.04). In the first evaluation of a clinic-based approach to address both RC and IPV in a low- or middle-income country (LMIC) context, we found evidence that ARCHES contraceptive counselling improved proximal outcomes related to contraceptive use and coping with RC and IPV. We recommend further study and refinement of this approach in Kenya and other LMICs.

**Plain Language Summary** Reproductive coercion (RC) and intimate partner violence (IPV) are two forms of gender-based violence that are known to harm women’s reproductive health. While one intervention, ARCHES – Addressing Reproductive Coercion in Health Settings, has shown promise to improve contraceptive use and help women cope with RC and IPV in the United States, no approach has been proven effective in a low- or middle-income country (LMIC) context. In the first evaluation of a reproductive empowerment contraceptive counselling intervention in an LMIC setting, we found that ARCHES contraceptive counselling, relative to standard contraceptive counselling, improved proximal outcomes on contraceptive uptake, covert contraceptive use, awareness of local violence survives, and reduced attitudes justifying RC among women seeking contraceptive services in Nairobi, Kenya. Distal outcomes will be reported separately. Findings from this study support the promise of addressing RC and IPV within routine contraceptive counselling in Kenya on women’s proximal outcomes related to contraceptive use and coping with violence and coercion and should be used to inform the further study of this approach in Kenya and other LMICs.

## Résumé

Cette étude a été entreprise pour évaluer les retombées d’une intervention de conseil contraceptif en vue de l’autonomisation reproductive (ARCHES) adaptée aux dispensaires privés de Nairobi, Kenya, sur les résultats proximaux de l’utilisation de contraceptifs et l’utilisation secrète d’une contraception, l’auto-efficacité, la sensibilisation et le recours à des services pour les victimes de violence exercée par un partenaire intime (VPI), ainsi que les attitudes justifiant la coercition reproductive et la VPI. Nous avons mené un essai contrôlé par grappes auprès de patientes de la planification familiale (*n* = 659) dans six dispensaires privés; les patientes ont été assignées de manière non aléatoire à ARCHES ou au groupe témoin à Nairobi et dans les environs. Les patientes ont complété des entretiens immédiatement avant (période de référence) et après (sortie) le traitement, puis au cours d’un suivi trois et six mois après. Nous avons utilisé la pondération par l'inverse de la probabilité d'être traité (IPTW) appliquée aux modèles structurels marginaux du deuxième degré pour estimer les conséquences du traitement à l’aide d’une approche modifiée de l’intention de traiter. Après l’IPTW, les femmes recevant des conseils ARCHES en matière de contraception étaient plus susceptibles que le groupe témoin de recevoir une méthode contraceptive à la sortie (86% contre 75%, *p* < 0.001) et affichaient une augmentation relative significativement plus élevée de la sensibilisation aux services de VPI entre la période de référence et le suivi à trois mois (bêta 0,84, IC à 95% 0,13, 1,55) et à six mois (bêta 0,92, IC à 95% 0, 1,84) et une diminution relative des attitudes justifiant la coercition reproductive depuis la période de référence jusqu’au suivi de six mois (bêta −0,34, IC à 95% −0,65, −0,04). Dans la première évaluation d’une approche clinique pour traiter à la fois la coercition reproductive et la VPI dans un pays à revenu faible ou intermédiaire, nous avons trouvé des données montrant que le conseil ARCHES en matière de contraception améliorait les résultats proximaux liés à l’utilisation de contraceptifs et à la gestion de la coercition reproductive et la VPI. Nous recommandons de réaliser des études complémentaires et d’affiner cette approche au Kenya et dans d’autres pays à revenu faible ou intermédiaire.

## Resumen

Este estudio se llevó a cabo para evaluar el efecto de una intervención de consejería anticonceptiva para el empoderamiento reproductivo (ARCHES, por sus siglas en inglés) adaptada para clínicas privadas en Nairobi, Kenia, en los resultados proximales de uso de anticonceptivos y uso encubierto, autoeficacia, conocimiento y uso de servicios para sobrevivientes de violencia de pareja íntima (VPI) y actitudes que justifican la coerción reproductiva (CR) y la VPI. Realizamos un ensayo controlado por grupos con mujeres pacientes de servicios de planificación familiar (*N* = 659) en seis clínicas privadas no asignadas aleatoriamente a ARCHES o control en y alrededor de Nairobi, Kenia. Las pacientes realizaron entrevistas inmediatamente antes (línea de base) y después (salida) del tratamiento y en el seguimiento a los tres y seis meses. Utilizamos probabilidad inversa de ponderación del tratamiento (IPTW) aplicada a modelos estructurales marginales de diferencia-en-diferencias marginal para evaluar el efecto del tratamiento aplicando un enfoque modificado de intención-de-tratar. Después de IPTW, las mujeres que recibieron consejería anticonceptiva ARCHES, relativas a los controles, eran más propensas a recibir un método anticonceptivo a la salida (86% c. 75%, *p* < 0.001) y vieron un amento relativo significativamente mayor en su conocimiento de servicios de VPI entre la línea de base y el seguimiento a los tres (beta 0.84, IC al 95% de 0.13, 1.55) y seis meses (beta 0.92, IC al 95% de 0, 1.84) y una disminución relativa de actitudes que justifican la CR entre la línea de base y el seguimiento a los seis meses (beta −0.34, IC al 95% de −0.65, −0.04). En la primera evaluación del enfoque clínico para abordar la CR y la VPI en el contexto de un país de bajos o medianos ingresos (PBMI), encontramos evidencia de que la consejería anticonceptiva ARCHES mejoró los resultados proximales relacionados con el uso de anticonceptivos y el abordaje de la CR y la VPI. Recomendamos estudiar más a fondo y afinar este enfoque en Kenia y en otros PBMI.

## Introduction

In 2020, one in four women of reproductive age in low- or middle-income countries (LMIC), as defined by the World Bank,^1^ had unmet need for contraception.^2^ Opposition to contraceptive use by others, primarily male partners and family members, remains a top contributor to unmet need, particularly in Sub-Saharan Africa.^3^ Reproductive coercion (RC) describes behaviours, usually inflicted by male partners or family members, that exemplify this opposition, including pregnancy-promoting and -preventing actions, to control women’s contraceptive use and fertility.^4,5^ Tactics for RC perpetration include contraceptive sabotage (e.g. hiding or tampering with a contraceptive method), pregnancy coercion (e.g. threats, force or pressure to promote pregnancy and/or stop contraceptive use), and abortion coercion (e.g. threats, force or pressure to limit access to safe abortion or to have an abortion against her wishes).^6,7^

RC is a form of violence, a violation of an individual’s right to control their own body^8^ and is highly associated with intimate partner violence (IPV) – physical, sexual, or emotional violence from a former or current intimate partner.^9^ Both forms of gender-based violence (RC & IPV) have harmful effects on reproductive health including unintended pregnancy.^10–13^ While studies suggest that approximately one in three women globally will experience IPV in her lifetime, studies on RC in LMICs are nascent.^14^ Current evidence has found that population-based lifetime prevalence of RC varies by context, but can be as high as 37% in family planning settings.^15^ Despite recommendations from the World Health Organization and other agencies to include IPV identification and support in routine women’s health services,^16^ no clinic-based models that address both RC and IPV have been tested and proven efficacious in LMIC settings.

In Kenya, approximately one in four women have unmet need for contraceptives^2^ and two in five women report violence from an intimate partner.^17^ Prior studies have documented the association between violence and unmet need for contraceptives in Kenya and regionally throughout sub-Saharan Africa.^18^ Evidence in Kenya shows that RC is a pervasive problem which, like IPV, severely and negatively impacts women’s reproductive health and autonomy.^6,19^ Prior qualitative research has also documented strong social and gender norms that prioritise male decision-making and limit female access to and use of contraceptive services in this context.^20^

The Addressing Reproductive Coercion in Health Settings (ARCHES) intervention was designed to help women and girls control their reproductive autonomy despite facing opposition or violence, by training existing contraceptive providers to offer education, screening and referral on RC and IPV universally to female patients within standard contraceptive counselling services.^21^ ARCHES was first developed and tested in the United States; across two cluster randomised controlled trials with over 4,000 participants, it was found to reduce odds of pregnancy coercion, as well as increase knowledge and use of IPV services, self-efficacy to use contraceptives covertly, and leaving an unsafe or unhealthy relationship.^22,23^ Based on these results, ARCHES was adapted to and tested in private community-based clinics run by Family Health Options of Kenya (FHOK), an International Planned Parenthood (IPPF) member affiliate, in the greater Nairobi area.^24^

This paper describes results from the cluster-controlled trial evaluating the effect of ARCHES-enhanced contraceptive counselling as adapted to private clinics in Nairobi, Kenya on proximal outcomes, including contraceptive use, awareness and use of IPV survivor services, and attitudes justifying RC and IPV. We hypothesised that ARCHES would increase (1) contraceptive uptake and use; (2) self-efficacy to use contraceptives in the face of RC; (3) covert use of contraceptives; (4) awareness and use of local IPV support services; and (5) reduce attitudes justifying IPV and RC, as compared to standard-of-care contraceptive counselling. Effects on distal outcomes, including IPV, RC and pregnancy are reported elsewhere [forthcoming].^25^ As the first adaptation and evaluation of ARCHES in an LMIC context, results will be utilised to further adapt and inform the potential scaling of ARCHES in Kenya and across LMICs.

## Methods

### Study design and setting

We conducted a parallel-group, prospective, non-randomised, cluster-control trial to evaluate the efficacy of the ARCHES model of contraceptive counselling relative to standard contraceptive counselling. The trial was conducted in six private primary-care clinics operated by a Kenya-based non-governmental organisation, Family Health Options of Kenya (FHOK). All FHOK clinics in the greater Nairobi area were enumerated and assigned in a 1:1 allocation ratio to intervention or control conditions based on FHOK’s assessment of feasibility; pair-matched allocation was attempted but not useable due to inaccurate facility-level data. Providers from intervention clinics offering counselling on contraceptive methods were trained on the integration of ARCHES strategies within standard contraceptive counselling. Control clinics received no additional training and continued to offer standard contraceptive counselling services. The trial included four points of data collection: baseline (immediately prior to receiving service), exit (immediately after receiving service before leaving the clinic), and three-month and six-month follow-up.

### Intervention

The Addressing Reproductive Coercion in Health Settings (ARCHES) intervention was developed and tested in the United States^21–23^ before being adapted to the Kenyan context in a collaborative process with the study team, participating health administrators and providers. Adaptation involved formative research with female clients and providers and participatory review of clinical protocols into standard operating procedures, training manuals, provider tools, and client education materials along with a two-month pilot and refinement of materials. Details about the adaptation process are reported elsewhere.^24^ ARCHES is implemented via training health providers to integrate strategies during contraceptive counselling to help women to control their contraceptive use and pregnancy decisions despite opposition from male partners or family members, and to cope with experiences of IPV and RC. These strategies include: (1) offering information on RC, types of methods that can be used discreetly in the face of opposition, and strategies on how to use such methods discreetly, (2) screening for experiences of RC and IPV with a supportive and validating response reaffirming her right to make decisions about her reproductive health and be in a non-violent relationship, (3) offering a warm referral over the phone to those disclosing IPV, and (4) regardless of disclosure, offering a palm-sized mini-booklet with information on contraceptive methods, RC, IPV, and contacts for local IPV support services, to read in the clinic or take home. ARCHES strategies are only provided when visual and auditory privacy is achieved; if privacy is not possible standard contraceptive counselling is delivered. In the Kenya adaptation, protocols were added including: (1) separating any accompanying male partners or family members from female patients prior to delivery of ARCHES, and (2) offering to the female patient that they can bring their male partner back to the clinic for more information on contraceptives if desired.^24^ The ARCHES package included three-day training manuals for facilitators and providers, training slides, and a modified contraceptive counselling flipchart with ARCHES strategies integrated. Training included education on RC, IPV, provider bias and women-centred care, ARCHES strategies, and peer-based practice via role plays with supervision and feedback. Patient education materials included the palm-sized mini booklet and a poster in waiting and counselling rooms. While providers are trained to offer ARCHES with all clients at all visits, the intervention was tested in a single session lasting between 10 and 40 minutes (depending on the nature of the clients contraceptive needs). Providers were not offered incentives for delivering ARCHES.

### Participants

**Figure 1.**
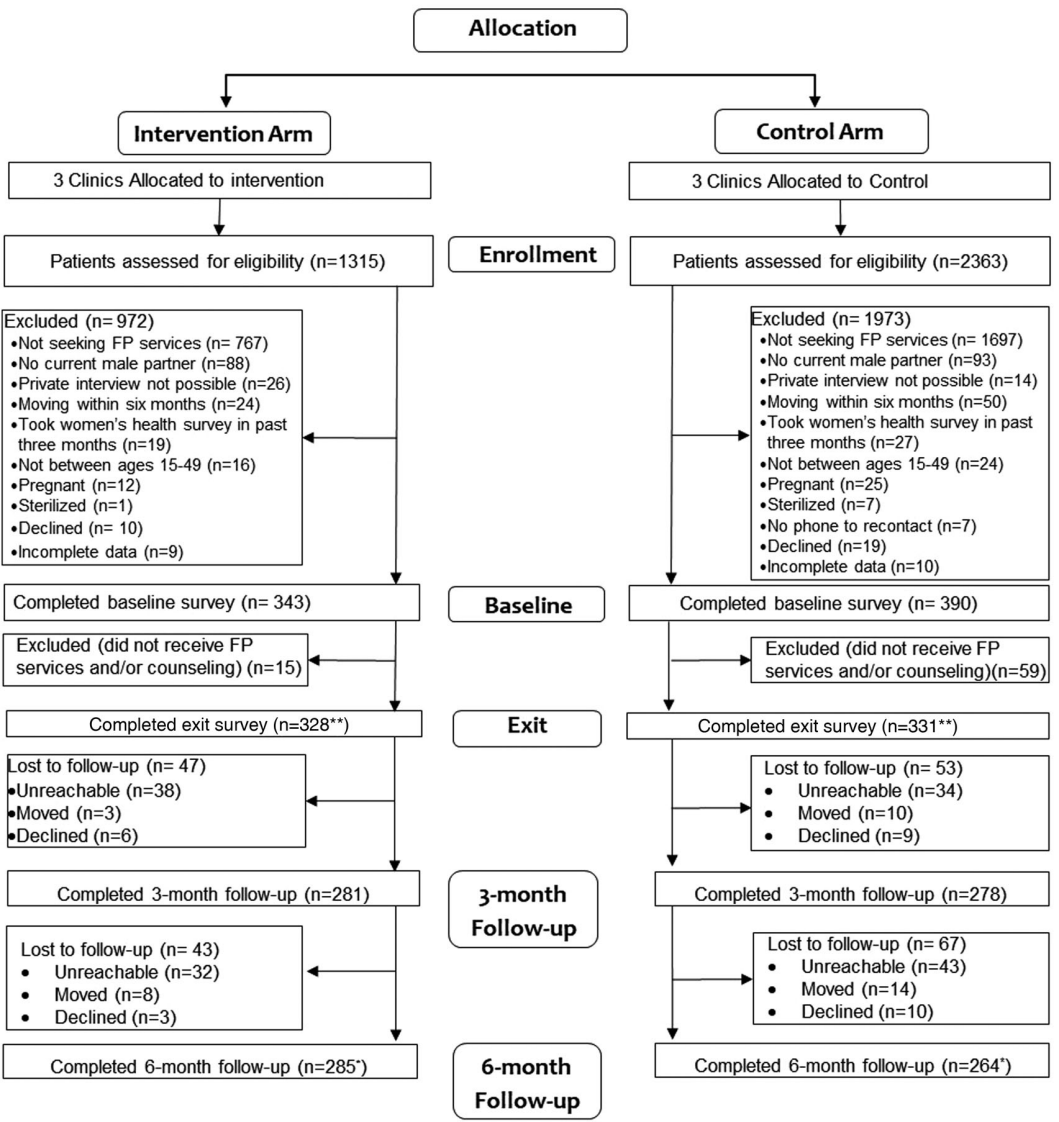
ARCHES cluster-controlled trial consort flow chart (Nairobi, Kenya; 2019) Notes: *Participants were contacted at the 6-month follow-up point irrespective of their participation in the 3-month follow-up, unless they had specifically declined further study participation. **Numbers at exit reflect those included in the analytical sample via the modified ITT approach

Participants were self-selected into intervention or control group based on the clinic in which they sought service. Participants were not blinded nor explicitly told which treatment group they were in, but may have been able to ascertain their treatment group based on the study description and services received. Female clients presenting at the clinic were asked by the receptionist if they were interested in participating in a “women’s health study”. Those interested were escorted by a trained research assistant to a private room in the clinic where they received information about the study, were screened for eligibility, and, if eligible and interested, completed written informed consent prior to baseline data collection. Women were eligible to participate in the study if they were “interested in receiving family planning services” at one of the six FHOK study clinics and self-reported being female, between the ages of 15-49, not currently pregnant or sterilised, having a male partner with whom they had sex in the past three months, no plans to move out of the area for the next six months, having a mobile phone safe for re-contacting for follow-up surveys, and being able to participate in a private interview. Because two trial facilities were previously pilot facilities, women who reported having taken a health survey in the past three months were excluded. Surveys were administered by research assistants using tablet computers in English or Kiswahili based on the participants’ preferences. Participants completed surveys at all time points at the clinic of enrolment. In a few cases, follow-up interviews were completed over the phone for those unable to return to the clinic. Patients were enrolled from July to December 2018 and follow-up from October 2018 to June 2019. The trial was stopped in June 2019 after the sample size was met and follow-up completed (Figure 1). No adverse events were reported.

### Measures

#### Outcomes

The primary proximal outcome reported in this study is uptake of a modern contraceptive method, assessed as a binary measure at exit where clients report having “received a family planning method today” from their provider including the IUD, implant, injection, contraceptive pills, or male/female condoms. Other distal primary outcomes are reported separately [forthcoming].^25^ Contraceptive use at three- and six-month follow-up is assessed as a binary measure based on self-report of currently using any of these modern contraceptive methods; the outcome is measured as change from baseline to three- and six-month follow-up.

Secondary proximal outcomes, assessed as the change from baseline to three- and baseline to six-month follow-up, include self-efficacy to use contraceptives in the face of RC, covert use of contraceptives, attitudes justifying RC and IPV, and awareness and use of local IPV survivor services (Supplement 1).

Self-efficacy to use contraceptives in the face of RC is assessed using three items on participants’ confidence in their ability to use a contraceptive method in the face of opposition (e.g. “How confident are you in your ability to use family planning if your partner tries to interfere?”). Participants respond on a three-point Likert scale. The variable is modelled as a complete-case sum score (range 0-6, Cronbach’s alpha = 0.75). Originally four items were asked; only three items were retained based on internal reliability assessments.

Covert use of contraceptives in the past three-months is assessed as a binary outcome based on participants’ response that they “used family planning without telling a male partner,” in the past three months.

Attitudes justifying RC are assessed via an eight-item scale where participants agree or disagree that it is appropriate for male partners to perpetrate forms of RC (e.g. “Is it acceptable for a male partner to force or pressure women to use family planning”) in a variety of situations (e.g. “if he wants more children than his partner”). Attitudes justifying IPV is assessed via a seven-item scale adapted from the Demographic and Health Survey wife-beating justification scale.^26,27^ Participants are asked to agree or disagree with statements on whether it was justified for a husband to beat their wife in a variety of situations (e.g. “she goes out without telling him”). Both variables are modelled as sum scores (1 = agree) with higher scores further justifying RC and IPV (range 0-8 for RC and 0-7 for IPV). Both scales demonstrated evidence of internal reliability in this sample (Cronbach’s alpha RC = 0.70, IPV = 0.78).

Awareness of local IPV survivor services is assessed as a binary outcome based on if participants “think a woman experiencing physical or sexual violence from her male partner could get help” from a list of four local IPV services. Participants who respond yes to at least one of the services are considered aware of local IPV survivor services. Utilisation of such services is also assessed as a binary outcome based on whether participants report having “called or visited” any of the services listed or any other services for IPV in the past three months.

#### Covariates

Covariates included as *a priori* hypothesised potential confounders include age (continuous), marital status (married or cohabitating, not married), highest education level attended (primary or less, secondary, tertiary or higher), parity (nulliparous, uniparous, multiparous), paid work in the past year, past 30-day food insecurity, language of survey administration (English, Swahili), and current modern contraceptive use at baseline (using, not using).

### Sample size

This study was designed to have 80% power to detect a 0.4 reduction in odds of intervention group reporting of RC in the past six months (equivalent to an 8-percentage point decrease) assuming a baseline prevalence of RC of 17%, cluster coefficient of variation of 0.5, inter-class correlation of <0.1%, and retention at three- and six-month follow-up visits of 85% and 75%, respectively, based on ARCHES studies completed in the United States.^22,23^ Power calculations were completed in STATA 14.

### Analyses

All analyses are conducted at the individual level. Descriptive statistics assessed client characteristics at baseline by treatment group and outcomes at each timepoint. Primary treatment effects were assessed via a modified intent-to-treat (mITT) approach where all patients who reported receiving any contraceptive counselling at the exit were included based on the treatment group assigned at enrolment. The modified ITT approach was used, given that a high proportion of clients originally enrolled in control clinics (15%) reported not receiving any contraceptive counselling. Secondary exploratory analyses were completed using an as-treated approach where patients originally assigned to the treatment group were moved to the control group if they did not report receiving the four core ARCHES strategies at exit.

At baseline, we found that intervention and control groups were significantly different across most measured covariates. Inverse probability of treatment weighting (IPTW) was applied^28^ to correct baseline group imbalances using a marginal structural modelling (MSM) approach.^29^ We used the average treatment effect on the treated (ATT) estimand where control observations are weighted to balance with unweighted treatment observations^30^ given that the intervention group reported higher rates of RC and IPV. After constructing and applying IPTW using all hypothesised covariates to create the treatment weights, treatment groups displayed no significant differences across covariates (Table 1, Supplement 2).

**Table 1.** Female family planning client characteristics at baseline by ARCHES modified intent-to-treat (mITT) treatment groups, unweighted (unbalanced) and IPTW (balanced) (Nairobi, Kenya; 2019)

Characteristic	Unweighted (*n* = 659)				mITT IPTW^b^ (*n* = 661.05)			
		Treatment group, *n* (%)				Treatment group, *n* (%)		
	Total Sample (*n* = 659)	ARCHES Intervention (*n* = 328)	Control (*n* = 331)	*p*^a^	Total Sample (weighted *n* = 661.05)	ARCHES Intervention (*n* = 328)	Control (weighted *n* = 333.05)	*p*^a^
Age, mean (std dev)	27.29 (7.27)	26.70 (6.63)	27.86 (7.81)	**0.04**	26.67 (6.69)	26.70 (6.63)	26.64 (6.77)	0.91
Married	429 (65.10)	230 (70.12)	199 (60.12)	**0**.**01**	457.67 (69.23)	230 (70.12)	227.67 (68.36)	0.62
Education level				**<0**.**001**				0.92
Primary or less	145 (22.00)	95 (28.96)	50 (15.11)		188.11 (28.46)	95 (28.96)	93.11 (27.96)	
Secondary	238 (36.12)	160 (48.78)	78 (23.56)		327.60 (49.56)	160 (48.78)	167.60 (50.32)	
Tertiary or higher	276 (41.88)	73 (22.26)	203 (61.33)		145.33 (21.99)	73 (22.26)	72.33 (21.72)	
Parity				**<0**.**001**				0.70
Nulliparous	147 (22.31)	46 (14.02)	101 (30.51)		85.91 (13.00)	46 (14.02)	39.91 (11.98)	
Uniparous	201 (30.50)	110 (33.54)	91 (27.49)		228.09 (34.50)	110 (33.54)	118.09 (35.46)	
Multiparous	311 (47.19)	172 (52.44)	139 (41.99)		347.04 (52.50)	172 (52.44)	175.04 (52.56)	
Food insecurity past 30 days	120 (18.21)	80 (24.39)	40 (12.08)	**<0**.**001**	175.22 (26.51)	80 (24.39)	95.22 (28.59)	0.22
Paid work past year	455 (69.04)	230 (70.12)	225 (67.98)	0.55	474.69 (71.81)	230 (70.12)	244.69 (73.47)	0.34
Language of survey administration				**<0**.**001**				0.59
English	192 (29.14)	66 (20.12)	126 (38.07)		138.73 (20.99)	66 (20.12)	72.73 (21.84)	
Swahili	467 (70.86)	262 (79.88)	205 (61.93)		522.32 (79.01)	262 (79.88)	260.32 (78.16)	
Current modern contraceptive use	490 (74.36)	263 (80.18)	227 (68.58)	**<0**.**01**	528.28 (79.92)	263 (80.18)	265.28 (79.65)	0.86

^a^ *P*-values are based on chi-square analyses for categorical variables and on Satterthwaite T-tests assuming unequal variances for continuous variables.

^b^ Adjusted for inverse probability of treatment weights (IPTW – weighted on treatment against baseline characteristics) using ATT (average effect among the treated) weighting where the control group is weighted to match the characteristics of the intervention group. Baseline characteristics used for weighting included age (continuous), marital status, education level, parity, food insecurity, language of survey administration, employment for paid work in the past year, and current modern contraceptive use.

**Figure 2.**
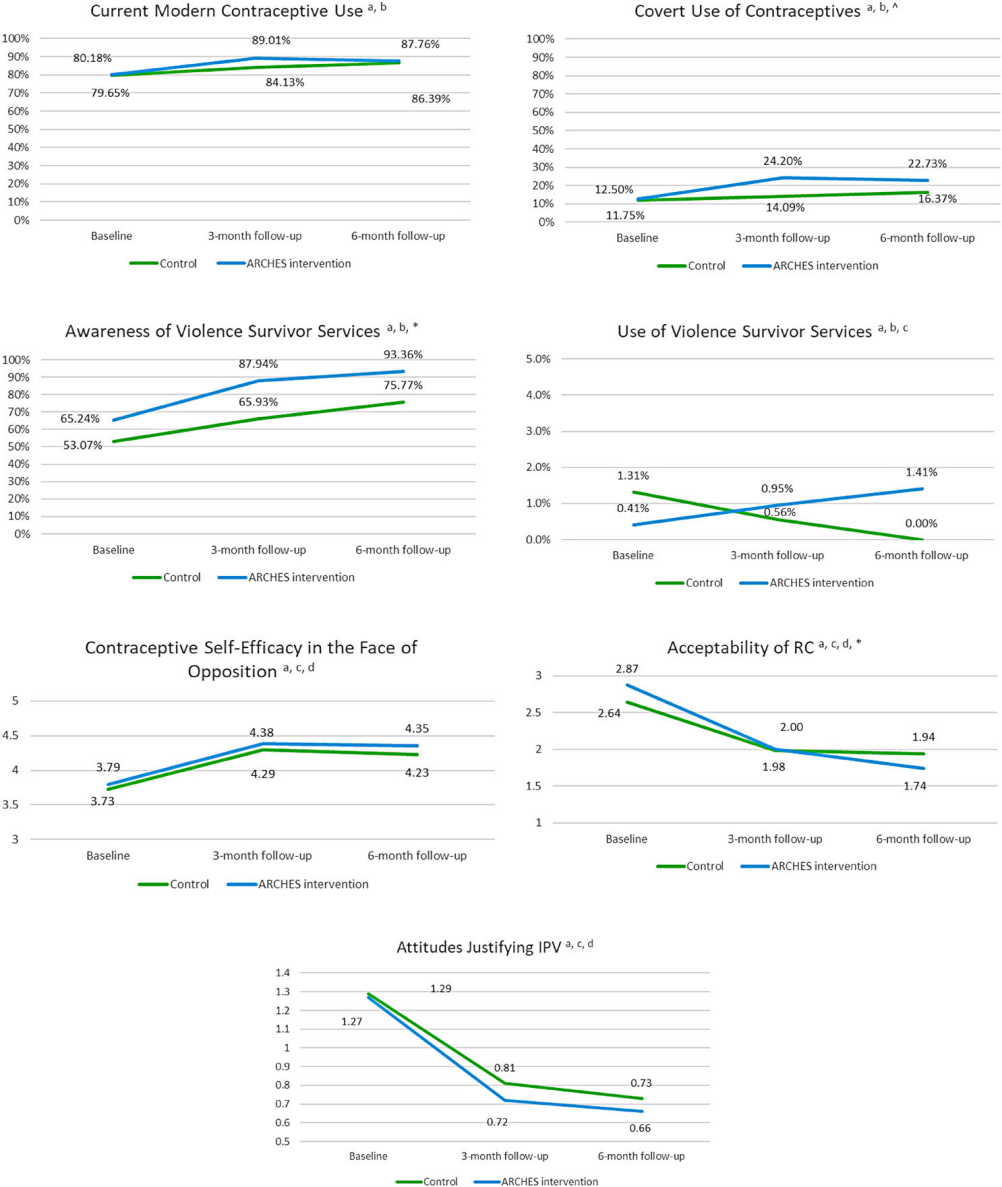
IPTW descriptive outcomes [proportions (binary) and means (continuous)] graphed over time within mITT treatment groups (Nairobi, Kenya; 2019) ^a^Reporting using inverse probability of treatment weights (IPTW - weighted on treatment against baseline characteristics) using ATT (average effect among the treated. Baseline characteristics used for weighting include age (continuous), marital status, education level, parity, food insecurity, language of survey administration, employment for paid work in the past year, and current modern contraceptive use. ^b^Binary outcome, reported in IPTW proportions ^c^Scale resized to improve readability ^d^Continuous outcome, reported in IPTW means Symbols are utilized to denote where the beta (i.e. average change in slope) between intervention and control at three- and/or six-month follow-up is statistically significant (see Table 3): ^p<.1, * p<.05

To assess time trends, bivariate IPTW treatment differences were estimated (Table 2). Bivariate tests were used to assess differences within treatment groups from baseline to three-month follow-up and from baseline to six-month follow-up. Chi-squared tests were utilised for binary outcomes (contraceptive uptake and use, covert contraceptive use, and awareness and use of local IPV services) and Satterthwaite T-tests assuming unequal variances were utilised for continuous outcomes (self-efficacy to utilise contraceptives in the face of RC, attitudes justifying RC and IPV). To visualise results, we also plotted the IPTW proportions (binary) and means (continuous) of each treatment group over time (Figure 2).

**Table 2.** IPTW descriptive outcomes at each time point within mITT treatment groups (Nairobi, Kenya; 2019)

	Control (weighted *n *= 333.05)^a^					ARCHES Intervention (*n* = 328)				
	Baseline	3-month follow-up	*p*-value^b^	6-month follow-up	*p*-value^c^	Baseline	3-month follow-up	*p*-value^b^	6-month follow-up	*p*-value^c^
Current modern contraceptive use, *n* (%)	265.28 (79.65)	242.63 (84.13)	0.15	233.40 (86.39)	**0.03**	263 (80.18)	251 (89.01)	**<**.**01**	251 (87.76)	**0**.**01**
Covert use of contraceptives, *n* (%)	39.12 (11.83)	40.40 (14.09)	0.40	44.23 (16.37)	0.11	41 (12.81)	68 (24.20)	**<**.**001**	65 (22.73)	**0**.**001**
Contraceptive self-efficacy in the face of opposition, mean (std)	3.73 (1.93)	4.29 (1.94)	**<**.**001**	4.23 (1.88)	**<**.**01**	3.79 (2.03)	4.38 (1.85)	**<**.**001**	4.35 (1.88)	**<**.**001**
Awareness of local violence survivor services, *n* (%)	176.75 (53.07)	188.01 (65.93)	**<**.**01**	204.72 (75.77)	**<**.**001**	214 (65.24)	248 (87.94)	**<**.**001**	267 (93.36)	**<**.**001**
Use of local violence survivor services, *n* (%)^d^	3.02 (1.31)	1.16 (0.56)	0.44	0	N/A	1 (0.41)	2 (0.95)	0.48	3 (1.41)	0.25
Acceptability of RC, mean (std)	2.64 (1.96)	1.98 (1.82)	**<**.**001**	1.94 (1.76)	**<**.**001**	2.87 (2.04)	2.00 (1.84)	**<**.**001**	1.74 (1.72)	**<**.**001**
Acceptability of IPV, mean (std)	1.29 (1.70)	0.81 (1.34)	**<**.**01**	0.73 (1.13)	**<**.**01**	1.27 (1.68)	0.72 (1.25)	**<**.**001**	0.66 (1.11)	**<**.**001**

^a^ Adjusted for inverse probability of treatment weights (IPTW – weighted on treatment against baseline characteristics) using ATT (average effect among the treated) weighting where the control group is weighted to match the characteristics of the intervention group. Baseline characteristics used for weighting include age (continuous), marital status, education level, parity, food insecurity, language of survey administration, employment for paid work in the past year, and current modern contraceptive use.

^b^ *P*-values for the within treatment group difference from baseline to 3-month follow-up are based on chi-square analyses for categorical variables and on Satterthwaite *T*-tests assuming unequal variances for continuous variables.

^c^ *P*-values for the within treatment group difference from baseline to 6-month follow-up are based on chi-square analyses for categorical variables and on Satterthwaite *T*-tests assuming unequal variances for continuous variables.

^d^ *P*-values based on Fisher's exact test due to small *n*.

Difference-in-differences (DiD) marginal structural regression models (MSM) were used to estimate the treatment effect comparing the relative change over time between intervention and control groups from baseline to three- and baseline to six-month follow-up (Table 3). DiD analysis is appropriate for estimating unbiased causal effects for non-randomised clinic-based interventions, as it separates the effect of unbalanced intervention and control groups at baseline, temporal trends in the outcome, and the impact of the intervention.^31,32^ We utilised multi-level mixed-effect logistic and linear DiD regression models with IPTW weights^28^ to assess the average treatment effect among the treated. DiD was used in combination with IPTW to identify the relative change over time as outcomes still showed differences between treatment groups at baseline after weighting for hypothesised confounders.^33^ Logistic regression was completed for binary outcomes and linear regression for continuous outcomes.^33^ Random effects were included to account for the within-group variation of the cluster (clinic) and repeated observations of individuals over time nested within clusters. Fixed effects were included for time, treatment, and the interaction of time by treatment.^31^ The beta and *p*-value of the time by treatment interaction term (both for linear and logistic regression) were used to estimate the treatment effect among the treated, relative to controls (Table 3). To aid in interpretation, we also calculated the odds ratios (binary/logistic) and betas (continuous/linear) comparing intervention to control at each time point (baseline, three- and six-month follow-up) from the time by treatment interaction terms (Supplement 3). No imputation was completed for missing data; only complete cases were analysed. For all testing, significance was defined as *p *≤ 0.05 and marginal significance as *p* < 0.1. All analyses were completed in SAS version 9.4.^34^

**Table 3.** Difference-in-differences average treatment effect among the treated (time × treatment) comparing treatment groups (ref = control) from multi-variable mixed-effect regression (Nairobi, Kenya; 2019)

Outcome	IPTW difference-in-differences (time × treatment) parameter ^a^ (95% confidence interval)			
	Baseline to 3-month follow-up	*p*-value	Baseline to 6-month follow-up	*p*-value
***mITT approach***				
Logistic regression [Beta – time × treatment (95% confidence interval)]^b^				
Current modern contraceptive use	0.43 (−0.19, 1.03)	0.17	0.13 (−1.05, 1.32)	0.83
Covert use of contraceptives	0.77 (−0.09, 1.63)	*0*.*08*	0.38 (−0.92, 1.69)	0.57
Awareness of local violence survivor services	0.84 (0.13, 1.55)	**0**.**02**	0.92 (0, 1.84)	**0**.**05**
Linear regression [Beta – time × treatment (95% confidence interval)]^b^				
Contraceptive self-efficacy in the face of opposition (Cronbach alpha = 0.75)	0.11 (−0.19, 0.42)	0.47	0.07 (−0.24, 0.37)	0.68
Acceptability of RC (Cronbach alpha = 0.70)	−0.12 (−0.42, 0.18)	0.44	−0.34 (−0.65, −0.04)	**0**.**03**
Acceptability of IPV (Cronbach alpha = 0.78)	−0.04 (−0.26, 0.18)	0.72	0.02 (−0.20, 0.24)	0.86
***As-treated approach***				
Logistic regression [Beta – time × treatment (95% confidence interval)]^b^				
Current modern contraceptive use	0.28 (−0.45, 1.01)	0.45	0.12 (−0.94, 1.17)	0.83
Covert use of contraceptives	0.61 (−0.15, 1.37)	0.12	0.35 (−0.54, 1.24)	0.44
Awareness of local violence survivor services	0.78 (0.05, 1.51)	**0**.**04**	0.96 (0.28, 1.63)	**<**.**01**
Linear regression [Beta – time × treatment (95% confidence interval)]^b^				
Contraceptive self-efficacy in the face of opposition (Cronbach alpha = 0.75)	0.01 (−0.30, 0.31)	0.99	−0.04 (−0.35, 0.27)	0.80
Acceptability of RC (Cronbach alpha = 0.70)	−0.36 (−0.67, −0.05)	**0**.**02**	−0.24 (−0.54, 0.07)	0.13
Acceptability of IPV (Cronbach alpha = 0.78)	0.03 (−0.18, 0.25)	0.75	0.10 (−0.12, 0.32)	0.37

^a^ Adjusted for inverse probability of treatment weights (IPTW – weighted on treatment against baseline characteristics) using ATT (average effect among the treated) weighting where the control group is weighted to match the characteristics of the intervention group. Baseline characteristics used for weighting include age (continuous), marital status, education level, parity, food insecurity, language of survey administration, employment for paid work in the past year, and current modern contraceptive use.

^b^ DiD estimator reported is the beta for the interaction term of time × treatment from linear or logistic multi-variable mixed effect regression. Null value on these parameters is equal to zero with positive values (>0) indicating positive time trend and negative values (<0) indicating negative time trend. Odds ratios comparing intervention and control at each time point are shown in Supplement 1.

### Ethics

This study was approved by the University of California San Diego’s Human Research Protections Program (Protocol 170084, February 7, 2017), the Population Council Institutional Review Board (Protocol 797, January 18, 2017) and the Kenyatta National Hospital-University of Nairobi Ethics and Research Committee (Protocol P945/12/2016, March 2, 2017). All participants completed written informed consent.

## Results

### Sample description

In total, 659 women and girls were enrolled into the study and received contraceptive services and/or counselling (*n* = 328 intervention, *n* = 331 control), 85% of eligible patients who were screened. Retention at six-month follow-up was 87% in the intervention arm and 80% in the control arm (Figure 1). Approximately 80% of women in the intervention group were currently using a contraceptive method compared to 69% in the control group. No significant differences were found in baseline covariates among those lost to follow-up. At baseline, participants were 27 years old on average (range 16–49). Most women were married (65%) and employed for paid work in the past year (69%). Approximately one-in-two women were multiparous, one-in-three uniparous, and one-in-four nulliparous. Education was relatively high, with 22% of women reporting tertiary or higher education. Less than one in five had reported food insecurity in the past 30 days. Participants receiving ARCHES, on average, had significantly lower levels of education and English survey administration and higher modern contraceptive use, food insecurity, and parity than the control group at baseline. Differences in covariates between treatment groups at baseline were not significant after IPTW, including baseline modern contraceptive use (Table 1, Supplement 2). Covariates did not vary significantly over time and no significant differences in covariates were found between those retained and those lost to follow-up. Intervention participants reported high quality of intervention implementation at exit; for each core element, study participants reported between 85% to 95% receipt and 77% of clients reported receiving all four core strategies. Of those intervention participants who reported RC or IPV on the baseline survey, over 70% reported disclosing this abuse to their ARCHES-trained provider and nearly all intervention participants (>98%) who were offered the mini booklet took it home.

### ARCHES effects on proximal outcomes

Patients who received ARCHES contraceptive counselling were more likely to report receiving a modern contraceptive method at their visit with the provider at exit than those receiving standard-of-care contraceptive counselling (75% among controls vs. 86% among ARCHES participants, *p* < 0.001, Supplement 4). The linear time trend for current modern contraceptive use increased significantly within the ARCHES treatment group at both three- and six-month follow-up (baseline to three-month follow-up 11% relative increase, *p* < 0.001; baseline to six-month follow-up 10% relative increase, *p* < 0.001) but only at six-month follow-up within the control group (baseline to three-month follow-up 5% relative increase, *p* = 0.15, baseline to six-month follow-up 7.5% relative increase, *p* = 0.03, Table 2). Contraceptive use was relatively high in this sample overall, and the ARCHES intervention group reported higher current contraceptive use across time points (Figure 2); however, this difference between treatment groups was not significant in DiD models and was more pronounced at three-month follow-up (mITT baseline to three-month follow-up beta 0.43; 95% CI −0.19, 1.03; *p* = 0.17, baseline to six-month follow-up beta 0.13; 95% CI −1.05, 1.32; *p* = 0.83, Table 3).

From baseline to three- and six-month follow-up, covert use of contraceptives increased significantly within the intervention group (baseline to three-month follow-up 94% relative increase, *p* < 0.001; baseline to six-month follow-up 82% relative increase, *p* < 0.001, Table 2). When compared to control in mITT DiD models, the ARCHES intervention group demonstrated a marginally significant increase in covert contraceptive use from baseline to three-month follow-up but not baseline to six-month follow-up (mITT baseline to three-month follow-up beta 0.77; 95% CI −0.09,1.63; *p* = 0.08, baseline to six-month follow-up beta 0.38; 95% CI −0.92, 1.69; *p* = 0.57, Table 3). In as-treated models the baseline to three-month follow-up had a similar magnitude but was not significant at *p* < 0.05 (as-treated baseline to three-month follow-up beta 0.61; 95% CI −0.15, 1.37; *p* = 0.12, baseline to six-month follow-up beta 0.35; −0.54, 1.24; *p* = 0.44, Table 3). While contraceptive self-efficacy in the face of opposition increased significantly over time within both control and intervention groups (Table 2) the change over time between groups was parallel (Figure 2). DiD regression models found no significant effect when comparing differences between treatment groups over time via the mITT or as-treated approaches (mITT baseline to three-month follow-up beta 0.11; 95% CI −0.19, 0.42; *p* = 0.47, baseline to six-month follow-up beta 0.07; 95% CI 0.68, 0.37; *p* = 0.68, Table 3).

Awareness of local IPV services increased significantly within both control (baseline to three-month follow-up 24% relative increase, *p* < 0.01; baseline to six-month follow-up 43% relative increase, *p* < 0.001) and intervention groups (baseline to three-month follow-up 35% relative increase, *p* < 0.001; baseline to six-month follow-up 43% relative increase, *p* < 0.001, Table 2) though awareness was higher in the intervention group at all time points (Figure 2). In DiD regression models, we found that the change over time in the intervention group was significantly greater than that of the control from baseline to both three- and six-month follow-up via both mITT and as-treated approaches (mITT baseline to three-month follow-up beta 0.84; 95% CI 0.13, 1.55; *p* = 0.02, baseline to six-month follow-up beta 0.92; 95% CI 0–1.84; *p* = 0.05, Table 3). Use of local violence survivor services was low in both control and ARCHES intervention groups, comprising less than 1% of the total sample at baseline (3% among those reporting current IPV); too small for testing.

Attitudes justifying IPV and RC decreased significantly at both follow-up time points in both ARCHES treatment and control groups (Table 2). For attitudes justifying IPV, this decrease over time was relatively similar for intervention and control (Figure 2) and we found no treatment effect in DiD regression models (mITT baseline to three-month follow-up beta −0.04; 95% CI −0.26,0.18; *p* = 0.72, baseline to six-month follow-up beta 0.02; 95% CI −0.20, 0.24; *p* = 0.86, Table 3). For RC, in the mITT approach, at baseline the ARCHES intervention group reported greater attitudes justifying RC than controls, but by six-month follow-up, the intervention group reported lower attitudes justifying RC than controls (Figure 2). mITT DiD regression models confirmed that women receiving the ARCHES intervention had a significantly greater reduction in attitudes justifying RC from baseline to six-month follow-up as compared to controls (mITT baseline to three-month follow-up beta −0.12; 94% CI −0.42, 0.18; *p* = 0.44, baseline to six-month follow-up beta −0.34; 95% CI −0.65, −0.04; *p* = 0.03, Table 3). As-treated DiD regression found that women receiving the ARCHES intervention had significantly greater reduction in attitudes justifying RC from baseline to three-month follow-up as compared to controls, but this effect was not significant from baseline to six-month follow-up (as-treated baseline to three-month follow-up beta −0.36; 95% CI −0.67, 0.05; *p* = 0.02, baseline to six-month follow-up beta −0.24; 95% CI −0.54, 0.07; *p* = 0.13, Table 3).

## Discussion

In the first evaluation of a clinic-based model to address RC and IPV within contraceptive counselling in an LMIC, this trial found evidence that ARCHES was implemented with relatively high fidelity by providers and increased contraceptive uptake at the appointment, covert contraceptive use, awareness of IPV services and decreased attitudes justifying RC over time as compared to standard contraceptive counselling among female patients seeking contraceptive services from private clinics in Nairobi, Kenya. We did not find an effect, however, of the intervention as compared to controls, in the change of contraceptive use overtime, contraceptive self-efficacy in the face of opposition, or attitudes justifying IPV overtime.

Women receiving ARCHES were more likely to have received a contraceptive method at their service visit than those in the control group. Prior studies have shown that improved quality of family planning services can increase contraceptive use^35,36^ including in one recent study in Kenya.^37^ Through ARCHES, providers are trained in women-centred care designed to prioritise women’s voice and goals for their contraceptive use and fertility which is expected to increase quality of care received, thus, we hypothesise, impacting contraceptive uptake. This is based on existing research that documents the links between person-centred care and improved perceived quality of reproductive health care.^38^ Further qualitative study on ARCHES’ effects on quality of care will be reported elsewhere [forthcoming].^25^

We also found evidence that ARCHES marginally increased covert use of contraceptives at three-month follow-up, but this was not maintained at six-month follow-up. Unlike in the United States, we found no significant effect on self-efficacy to use a contraceptive method in the face of RC, including using covertly. Covert contraceptive use is an act of female resistance in the face of restrictive, patriarchal gender norms that prioritise male-decision-making on family planning^39,40^ and emerging studies show women reporting RC and IPV are more likely to use contraceptives covertly.^40^ Recent research, however, has also documented challenges of covert use over time such as emotional distress due to fear of their partner finding out and difficulties hiding side effects.^41^ It is possible that such challenges impacted ARCHES clients’ self-efficacy to use methods covertly. While findings on covert use at three-month follow-up were marginally significant, diagram representations of results (Figure 2) support a likely effect of ARCHES on increases in covert use of contraceptives that we were underpowered to detect at alpha <0.05. Larger longitudinal studies are required to clarify the effect of ARCHES on covert contraceptive use, the efficacy of covert use to prevent unintended pregnancy, and potential risks associated with contraceptive use and exposure to violence.

Like findings from the evaluation in the United States,^22^ women receiving ARCHES contraceptive counselling had increased odds of being aware of local IPV services compared to women receiving standard-of-care contraceptive counselling; however, almost no clients sought IPV care. This aligns with previous studies showing low utilisation of IPV survivor services due to limited availability of conveniently accessible services (leading to high cost and time required to access available services), shame, and concern about confidentiality and stigmatisation, barriers which may be particularly salient in LMICs.^10,42-44^ Studies indicate the promise of improving IPV service utilisation in LMICs by adding integrated referral structures at the facility level, including increased training on first line IPV response, increased number of providers trained on IPV first-line support, and established referral linkage to higher-level care, and linking clinic-based care with community-based social support structures.^45,46^ This adaptation of ARCHES focused on increasing the number of providers trained on basic first-line response at the facility level and linkage to higher-level care at offsite facilities, however, additional approaches may be required to increase utilisation.

We also found that ARCHES reduced attitudes justifying RC over time as compared to those receiving standard of care, and that both the intervention and control group saw a significant decrease in attitudes justifying IPV. This similar decrease in attitudes justifying IPV, as well as comparable increases in self-efficacy to use contraceptives in the face of RC, may be explained by the survey acting as an intervention. Other studies have shown that simply asking the survey questions may cause a testing effect.^47–49^ Despite this possibility, we did find that ARCHES resulted in a significantly greater reduction in attitudes justifying RC over time as compared to the control. Decreased acceptance of abuse can be a first step in help-seeking and coping with or leaving abusive relationships^42^ while attitudes justifying abuse can normalise violence.^50^ Results from this study support the importance of providing opportunities to disclose both RC and IPV with supportive provider response within routine contraceptive care.

This study has several limitations which highlight opportunities for further study. The evaluation was conducted among women visiting six private clinics in metropolitan Nairobi. Our sample is not representative of women seeking contraceptive care across Kenya or in public facilities in Nairobi, thus, results cannot be generalised. Future studies should consider testing the approach among a more representative population. Additionally, lack of randomisation and the small number of clusters created imbalanced groups at baseline. While this was adjusted for using IPTW and DiD, it is possible that unmeasured confounders remained. For example, it is possible that the effect on contraceptive uptake was the result of unmeasured confounding by the type of family planning service sought. Larger randomised controlled trials with additional possible confounder measurement are needed to clarify and confirm effects found in this study. Self-report data, particularly on violence and coercion, is subject to social desirability and testing biases which may have differed across groups based on the intervention. Furthermore, the intervention itself is limited to reaching only those women who have the privilege and access to seek contraceptive care. It is unknown if the main strategies of the intervention would have an impact outside of this setting, and, while this does not diminish the need for interventions to address the common experiences of violence and coercion in clinical settings, it does highlight the need for further adaptation of these intervention approaches in the community context to reach those most at need. Finally, the evaluated approach was resource-intensive, requiring a three-day provider training, which may not be sustainable at scale. Future work is needed to refine the approach into a scalable model and to re-test intervention effectiveness within a less resource-intensive approach. Additionally, cost-effectiveness studies are needed to clarify for policy makers the practical cost-benefit of the intervention. Despite these limitations, this study offers valuable learnings on proximal outcomes for interventions striving to improve women’s reproductive autonomy and address IPV within routine contraceptive care in Kenya and similar contexts and underscores pathways for future research.

## Conclusion

In this initial evaluation of a clinic-based approach to address both RC and IPV in an LMIC setting, we found evidence that the ARCHES model of contraceptive counselling improves contraceptive uptake and awareness of IPV services and reduces attitudes justifying RC in comparison to standard contraceptive care. Based on these results, we recommend further study of this approach in Kenya and other LMIC contexts.

## Supplementary Material

ARCHES proximal supplements 1-4

## Data Availability

Statistical code and the de-identified analysis dataset is available from the Dryad repository, DOI https://doi.org/10.6076/D1Q30G.
