# Supplementary material for: Effects of a clinic-based reproductive empowerment intervention on proximal outcomes of contraceptive use, self-efficacy, attitudes, and awareness and use of survivor services: a cluster-controlled trial in Nairobi, Kenya
Source: Sex Reprod Health Matters. 2023 Aug 18;31(1):2227371. doi: 10.1080/26410397.2023.2227371 (PMC10443967; doi:10.1080/26410397.2023.2227371)
Supplement: ARCHES proximal supplements 1-4 [file ZRHM_A_2227371_SM7461.docx]

Supplement 1. Outcome measure survey items

| Construct | Interview Question(s) | Response Options |
| --- | --- | --- |
| Uptake of modern contraceptive method at exit | Did you receive a family planning method today from your provider? | Yes  No |
|  | What method did you receive? | IUD?  Injection?  Implant?  Pill?  Male or female condom?  Other method? |
| Current modern contraceptive use | Are you currently using a family planning method? | Yes  No |
|  | Which of these family planning method(s) are you currently using? | IUD?  Injection?  Implant?  Pill?  Male or female condom?  Other method? |
| Self-efficacy to use contraceptives in the face of RC | How confident are you in your ability to use family planning even if your partner tries to interfere? | Very confident  Somewhat confident  Not at all confident |
|  | How confident are you that you could use family planning without your partner knowing? | Very confident  Somewhat confident  Not at all confident |
|  | If your partner stopped you from using a family planning method, how confident are you that you could switch to a different family planning method and use it without him knowing? | Very confident  Somewhat confident  Not at all confident |
| Covert use of contraceptives | Does your current partner know that you are using your current method of family planning? | Yes  No |
| Attitudes justifying RC | Some husbands/partners force or pressure women to become pregnant or make it difficult for them to use family planning.  This is acceptable if…  he wants more children than his partner. | Agree  Disagree |
|  | they haven't had any children. | Agree  Disagree |
|  | She hasn't had any sons. | Agree  Disagree |
|  | It is acceptable for a husband/partner to stop a woman from using family planning if she is experiencing side effects he does not like (e.g. being cold, not interested in sex). | Agree  Disagree |
|  | It is acceptable for a husband/partner to stop a woman from using family planning if he is worried about her having an affair/having sex with other men. | Agree  Disagree |
|  | It is acceptable for a husband/ male partner to throw away, destroy, hide, or otherwise interfere with a woman's family planning method. | Agree  Disagree |
|  | It is acceptable for a husband/partner to leave his wife for refusing to become pregnant if he wants more children than his partner. | Agree  Disagree |
|  | In your opinion, it is acceptable for a husband/partner to have children with another woman if his current partner refuses to get pregnant. | Agree  Disagree |
| Attitudes justifying IPV | A husband is justified in beating his wife if…  she goes out without telling him. | Agree  Disagree |
|  | she neglects the children. | Agree  Disagree |
|  | she argues with him. | Agree  Disagree |
|  | she refuses to have sex with him. | Agree  Disagree |
|  | she burns the food. | Agree  Disagree |
|  | she refuses to get pregnant. | Agree  Disagree |
|  | she uses family planning without his knowledge. | Agree  Disagree |
| Awareness of local IPV services | Do you think a woman experiencing physical or sexual violence from her male partner could get help at the following agencies or services?  Gender Based Violence Resource Center (GBVRC) at Kenyatta National Hospital? | Yes  No  Never heard of this service |
|  | Médecins Sans Frontières (MSF) Clinics (e.g. Lavender Clinic)? | Yes  No  Never heard of this service |
|  | Gender Violence Recovery Center (GVRC) at Nairobi Women's Hospital? | Yes  No  Never heard of this service |
|  | Gender Based Violence (GBV) Hotline? | Yes  No  Never heard of this service |
| Use of IPV services | Have you ever visited any of these agencies or services for physical and/or sexual violence from a male partner? | Yes  No |
|  | When was the last time that you visited one of these agencies or services?  Would you say… | Within the last month  Within the last three months  Within the last six months  Within the last twelve months  Longer than twelve months ago |

Supplement 2. Female family planning client characteristics at baseline by ARCHES as-treated treatment groups, unweighted (unbalanced) and IPTW (balanced) (Nairobi, Kenya; 2019).

| Characteristic | Unweighted | | | | As-treated IPTW^b^ | | | |
| --- | --- | --- | --- | --- | --- | --- | --- | --- |
|  |  | Treatment group, #(%) | |  |  | Treatment group, #(%) | |  |
|  | Total Sample  (n=659) | Intervention (n=254) | Control (n=405) | p^a^ | Total Sample  (n=510.66) | Intervention (n=254) | Control (n=256.66) | p^a^ |
| Age, mean (std dev) | 27.29 (7.27) | 26.51 (6.57) | 27.77 (7.64) | **0.03** |  | 26.51 (6.57) |  |  |
| Married | 429 (65.10) | 177 (69.69) | 252 (62.22) | **0.05** | 355.01 (69.52) | 177 (69.69) | 178.01 (69.36) | 0.94 |
| Education level |  |  |  | **<.0001** |  |  |  | 0.94 |
| Primary or less | 145 (22.00) | 77 (30.31) | 68 (16.79) |  | 152.64 (29.89) | 77 (30.31) | 75.64 (29.47) |  |
| Secondary | 238 (36.12) | 122 (48.03) | 116 (28.64) |  | 249.30 (48.82) | 122 (48.03) | 127.30 (49.60) |  |
| Tertiary or higher | 276 (41.88) | 55 (21.65) | 221 (54.57) |  | 108.72 (21.29) | 55 (21.65) | 53.71 (20.93) |  |
| Parity |  |  |  | **<.001** |  |  |  | 0.83 |
| Nulliparous | 147 (22.31) | 35 (13.78) | 112 (27.65) |  | 66.03 (12.93) | 35 (13.78) | 31.03 (12.09) |  |
| Uniparous | 201 (30.50) | 91 (35.83) | 110 (27.16) |  | 187.35 (36.69) | 91 (35.83) | 96.35 (37.54) |  |
| Multiparous | 311 (47.19) | 128 (50.39) | 183 (45.19) |  | 257.28 (50.38) | 128 (50.39) | 129.28 (50.37) |  |
| Food insecurity past 30 days | 120 (18.21) | 56 (13.83) | 64 (25.20) | **<.001** | 129.67 (25.39) | 56 (13.83) | 65.67 (25.59) | 0.92 |
| Paid work past year | 455 (69.04) | 179 (70.47) | 276 (68.15) | 0.53 | 361.65 (70.82) | 179 (70.47) | 182.65 (71.16) | 0.86 |
| Language of survey administration |  |  |  |  |  |  |  | 0.73 |
| English | 192 (29.14) | 46 (18.11) | 146 (36.05) | **<.0001** | 95.60 (18.72) | 46 (18.11) | 49.60 (19.32) |  |
| Swahili | 467 (70.86) | 208 (81.89) | 259 (63.95) |  | 415.06 (81.28) | 208 (81.89) | 207.06 (80.68) |  |
| Current modern contraceptive use | 490 (74.36) | 206 (81.10) | 284 (70.12) | **<.01** | 414.23 (81.12) | 206 (81.10) | 208.23 (81.14) | 0.99 |
| ^a^ P-values are based on chi-square analyses for categorical variables and on Satterthwaite T-tests assuming unequal variances for continuous variables. ^b^ Adjusted for inverse probability of treatment weights (IPTW - weighted on treatment against baseline characteristics) using ATT (average effect among the treated) weighting where the control group is weighted to match the characteristics of the intervention group. Baseline characteristics used for weighting included age (continuous), marital status, education level, parity, food insecurity, language of survey administration, employment for paid work in the past year, and current modern contraceptive use. | | | | | | | | |

Supplement 3. Differences in outcomes between treatment groups at each time point from multi-variable mixed-effect regression (Nairobi, Kenya; 2019).

| Outcome | Comparisons of Intervention to Control at Each Time Point ^a^ with IPTW ^b^  (95% confidence interval) | | | | | |
| --- | --- | --- | --- | --- | --- | --- |
|  | Baseline | p-value | 3-month Follow-up | p-value | 6-month  Follow-up | p-value |
| ***mITT approach*** |  |  |  |  |  |  |
| Logistic regression (Odds ratio – OR comparing treatment groups at each time point) | | | | | | |
| Current modern contraceptive use | 0.94 (0.31, 2.86) | 0.92 | 1.13 (0.49, 2.60) | 0.77 | 1.07 (0.39, 2.99) | 0.89 |
| Covert use of contraceptives | 1.65 (1.17, 2.33) | **0.01** | 2.41 (0.99, 5.83) | **0.05** | 2.41 (0.49, 11.87) | 0.28 |
| Awareness of local violence survivor services | 1.52 (0.41, 5.63) | 0.53 | 2.73 (0.78, 9.51) | 0.11 | 3.80 (1.01, 14.30) | **0.05** |
| Linear regression (Beta – comparing treatment groups at each time point) | | | | | | |
| Contraceptive self-efficacy in the face of opposition (alpha = 0.75) | 0.31 (-0.97, 1.60) | 0.63 | 0.37 (-0.91, 1.65) | 0.57 | 0.38 (-0.91, 1.67) | 0.57 |
| Acceptability of RC (alpha = 0.70) | 0.17 (-0.94, 1.28) | 0.77 | 0.01 (-1.09, 1.12) | 0.98 | -0.18 (-1.29, 0.94) | 0.76 |
| Acceptability of IPV (alpha = 0.78) | 0.10 (-0.60, 0.80) | 0.78 | 0.09 (-0.60, 0.78) | 0.79 | 0.12 (-0.58, 0.82) | 0.74 |
| ***As-treated approach*** |  |  |  |  |  |  |
| Logistic regression (Odds ratio – OR comparing treatment groups at each time point) | | | | | | |
| Current modern contraceptive use | 0.70 (0.33, 1.46) | 0.34 | 0.79 (0.55, 1.14) | 0.21 | 0.78 (0.41, 1.47) | 0.44 |
| Covert use of contraceptives | 0.87 (0.74, 1.02) | *0.08* | 1.19 (0.75, 1.89) | 0.46 | 1.23 (0.53, 2.82) | 0.63 |
| Awareness of local violence survivor services | 0.54 (0.37, 0.79) | **<.01** | 0.96 (0.59, 1.56) | 0.86 | 1.39 (0.81, 2.39) | 0.23 |
| Linear regression (Beta – comparing treatment groups at each time point) | | | | | | |
| Contraceptive self-efficacy in the face of opposition (alpha = 0.75) | 0.17 (-0.24, 0.59) | 0.41 | 0.16 (-0.23, 0.55) | 0.41 | 0.13 (-0.30, 0.57) | 0.55 |
| Acceptability of RC (alpha = 0.70) | 0.33 (-0.09, 0.74) | 0.12 | 0.13 (-0.25, 0.51) | 0.52 | 0.09 (-0.34, 0.51) | 0.68 |
| Acceptability of IPV (alpha = 0.78) | 0.22 (-0.10, 0.53) | 0.17 | 0.26 (-0.03, 0.55) | *0.08* | 0.32 (-0.01, 0.64) | **0.05** |
| ^a^ Estimated betas and odds ratios for the comparison of intervention to control at each time point. Estimated by setting the time point in the time x treatment interaction term and comparing the control vs. ARCHES intervention treatment groups. Odds ratios (exponentiated betas) are used for logistic regression outcomes and betas only for linear regression outcomes. ^b^ Adjusted for inverse probability of treatment weights (IPTW - weighted on treatment against baseline characteristics) using ATT (average effect among the treated) weighting where the control group is weighted to match the characteristics of the intervention group. Baseline characteristics used for weighting include age (continuous), marital status, education level, parity, food insecurity, language of survey administration, employment for paid work in the past year, and current modern contraceptive use. | | | | | | |

Supplement 4. Contraceptive uptake immediately post intervention (i.e., at exit) measuring the difference between treatment groups (Nairobi, Kenya; 2019).

| Outcome | IPTW ^a^ | | |
| --- | --- | --- | --- |
|  | Control  n, (%) | Intervention n, (%) | Chi-squared p-value ^b^ |
| ***mITT approach*** | | | |
| Received any modern FP method at appointment | 250.83 (75.31) | 282 (85.98) | **<.001** |
| ***As-treated approach*** | | | |
| Received any modern FP method at appointment | 200.50 (78.12) | 217 (85.43) | **0.03** |
| ^a^ Adjusted for inverse probability of treatment weights (IPTW - weighted on treatment against baseline characteristics) using ATT (average effect among the treated) weighting where the control group is weighted to match the characteristics of the intervention group. Baseline characteristics used for weighting include age (continuous), marital status, education level, parity, food insecurity, language of survey administration, employment for paid work in the past year, and current modern contraceptive use. ^b^ Chi-square statistics used where cells not less than n=5, otherwise, fisher-exact two-sided p-value reported. | | | |
